# Perspectives of Migrant Youth, Parents and Clinicians on Community-Based Mental Health Services: Negotiating Safe Pathways

**DOI:** 10.1007/s10826-017-0700-1

**Published:** 2017-03-27

**Authors:** Lucie Nadeau, Annie Jaimes, Janique Johnson-Lafleur, Cécile Rousseau

**Affiliations:** 1 Divisions of Social and Cultural Psychiatry and of Child Psychiatry, Department of Psychiatry, McGill University, Montreal, QC Canada; 2 Transcultural Research and Intervention Team (TRIT), CIUSSS du Centre-Ouest-de-l′île-de-Montréal, Montreal, QC Canada; 3 Department of Psychology, Université du Québec à Montréal (UQAM), Montreal, QC Canada

**Keywords:** Youth mental health services, Children and adolescents, Collaborative care, Patient perspective, Migrants

## Abstract

Youth mental health (YMH) services are greatly underutilized, particularly for migrant youth. Collaborative models of care offer promising avenues, but research on these treatment modalities is still scarce, particularly for migrants. The goal of this exploratory study is to better understand quality of care including factors improving access to care and collaborative YMH services use, efficacy and satisfaction, for this vulnerable population. This qualitative study relies on a multi-informants (youth, parents, clinicians) and multiple case study design to explore YMH collaborative services for migrant youth living in an urban setting (Montreal, Canada). Participants are five young patients (12–15 years old), one of their parents and their primary care therapist (*N* = 15). They come from migrant families, have a psychiatric diagnosis and have been receiving mental health services in a collaborative care setting for at least 6 months. Transcripts of semi-structured interviews for the five triads were thematically analyzed to draw similarities and contrasts between actors, across and within case-studies. Based on these findings, four themes emerged concerning the optimal care setting for collaborative YMH services for migrant families: (1) providing an equilibrium between communication, collaboration and privacy/confidentiality, (2) special attention to ensuring the continuity of care and the creation of a welcoming environment where trusting relationships can develop, (3) the inclusion of family intervention, and (4) the provision of collaborative decision-making pathways to care, addressing interprofessional and interinstitutional collaboration as well as cultural differences in explanatory models and values.

## Introduction

The accessibility and adequacy of youth mental health (YMH) services are ongoing concerns, as the number of youths with mental health issues who actually receive services is alarmingly low (Leckman and Leventhal 2008; McGorry et al. 2013). In our globalized societies, this is an even greater issue for migrant (immigrant or refugee) families not familiar with the health system in their new host country. Such families and their youth are known to underutilize mental health services and to face barriers to accessing these services (Ellis et al. 2011; Nadeau and Measham 2006; Ter Kuile et al. 2007). In addition, little attention has been paid to service provision in migrant and multiethnic neighborhoods, where the acceptability of mental health services may be a more complex issue and call for greater service adaptation (Nadeau and Measham 2006; Pina and Gonzales 2014). Cultural representations and recent migratory experiences influence the expression of mental health issues and the ways families seek help (Rousseau et al. 2007). The way mental health suffering is transformed into symptoms is framed by culture (Obeyesekere 1990) and socially sanctioned idioms of distress (Rechtman 2000). Explanatory models of mental health issues vary around the world, and migrants’ frames of reference regarding the reasons for the appearance of symptoms and the actions needed/expected to address such symptoms may differ from those held by their health care providers (Kleinman 1988). Moreover, migrants also go through a “cultural transition process” (Pumariega et al. 2005) as they interact with the host society, by which their representations of illness and treatment are transformed.

Collaborative care between mental health specialists and primary care clinicians is aimed at providing avenues for improving access to mental health care. Emerging research supports the advantages offered by this model in addressing barriers to care for vulnerable migrant youth (Rousseau et al. 2013). Collaborative care is a patient-centered model of services in which the patient is seen as a partner in decision-making; this model brings together all the professionals involved in care to elaborate a care plan and implement it (Kates et al. 2011). Collaborative models of services in YMH are complex, requiring a comprehensive intervention paradigm with multidisciplinary care and intersectoral service collaboration. Treatment needs vary according to the child’s development, clinical presentation, family issues, as well as co-contributing community and school factors in addition to the larger socio-cultural context. While specific models have been developed to operationalize collaborative care in YMH (Aupont et al. 2013; Kolko et al. 2012; Lipton and Donsky 2012; Nadeau et al. 2012), collaborative models still lack robust assessment to ensure optimal adaptation to YMH, a challenge that must be addressed through solid research-practice partnerships (Garland and Brookman-Frazee 2015).

In addition to the dearth of studies on the best models of care for YMH services (Tylee et al. 2007), especially for migrant families, there is little understanding of the patients’ perspectives (Gampetro et al. 2012; McGuirk and Button 2013) likely to inform these models, despite a movement toward patient-centered care and growing evidence that patient preference regarding treatment increases adherence and promotes better outcomes (McHugh et al. 2013). A more nuanced perspective could be achieved through the triangulation of clinician and family voices, which would be vital to understanding perceived barriers to care (Kazdin et al. 1997).

In order to further the field of YMH collaborative care models, the current study draws on underresearched elements important to consider when developing such models, especially for migrant youth. This project was designed in Montreal, Canada, to explore youth, parent and clinician perspectives on YMH services in a collaborative care model. The study took place in a primary-care, community-based health and social service center (CSSS) (Montréal, Canada) that serves a multiethnic population and has three local services centers (known as CLSCs). These centers have a tradition of offering proximity services in multiple locations (such as the CLSCs themselves, schools, or patients’ homes). Since 2007, YMH services have been offered as part of a collaborative care model involving multidisciplinary teams and on-site child psychiatrists acting as consultants and providing support to CSSS youth teams (in an integrated co-location model). This model entails the involvement of child psychiatrists in team meetings, as well as in formal and informal case discussions apart from consultations with patients at which main case workers are present. This exploratory study was aimed at developing a better understanding of the quality of care for migrant families including the process of accessing care and receiving collaborative YMH services as well as the efficiency of such services and the degree of satisfaction with them. The study employed a primarily qualitative research design, also incorporating the testing of quantitative questionnaires to be used in a wider study currently under way. The present article focuses on the project’s qualitative results. The study was the first step in a research program on collaborative YMH care, and provided an opportunity to document important themes and patterns in care trajectories which could potentially inform services and further research. A “follow the thread” research process was adopted to ensure these early results inform subsequent research steps (O’Cathain et al. 2010).

## Method

### Participants

The research project was presented to primary care clinicians during youth teams meetings at the community-based health and social service center. Staff members were invited to refer families to the research team. Families who were willing to be contacted then received a phone call from a research assistant who explained the study in further detail. This both allowed for a personalized approach to families through trusted service providers while clearly distinguishing between clinical work and research. Parents and youths were provided assurances that their participation in the study would not determine their current or future treatment. Each recruited youth’s main primary care clinician was invited to participate in the research, and all accepted. These clinicians were members of either the YMH team or the Youth in Difficulty team at the CLSC. The clinicians of these teams have recognized expertise in youth emotional and behavioral problems. They were informed that their participation would not have a foreseeable impact on their work. Inclusion criteria for youths were: being 12–17 years old, coming from a migrant family (either the family or parents having immigrated), and having received mental health services in a collaborative setting for at least 6 months. The study focused primarily on youth whose two parents had both immigrated, but also included one triad in which only one parent had immigrated as a contrasting experience. Youth and family members were offered compensation for their time (a $10 gift certificate or $20, respectively). Therapists were not compensated but interviews were held during working hours. Although the study’s objective was to use a purposeful sampling, challenges encountered in recruiting migrant participants resulted in the sample being only partly purposeful (Patton 2002). As planned, the sample included youth, parents and clinicians of both genders, some range in youth age, a diversity of professional backgrounds among clinicians, and families with one and two immigrant parents. The results suggest that, despite the aforementioned limitation, a range of opinions was expressed and high consensus was obtained on certain themes. Several emerging themes were able to surface as well. This suggests that the most commonly held opinions among our target populations were probably obtained through the 15 interviews, whereas a larger sample of triads might have allowed less prevalent themes to emerge as well. Ethical approval was obtained from the participating institution (CSSS de la Montagne). Parent and youth demographics and clinical portraits appeared to be representative of the population served by the clinic and are shown in Table 1.

**Table 1:** Description of study participants (demographic information and description of the emotional and behavioral difficulties)

Youth (*N* = 5)	Gender	4 boys
		1 girl
	Age	12, 13, 14, 14 and 15 years old
	Emotional and behavioral difficulties	4 youths with emotional external behavior problems
		1 youth with depression
		1 youth with Attention Deficit Hyperactivity Disorder
Parent (*N* = 5)	Gender	4 mothers
		1 father
	Ethnicity	3 families with both parents from South Asia
		1 family with both parents from Southeast Asia
		1 family with mixed couple of European and Canadian origin
	SES	4 families with low socioeconomic status
		1 family with medium socioeconomic status
Clinician (*N* = 5)	Gender	4 women
		1 man
	Profession	3 social workers
		1 creative art therapist
		1 psychoeducator

### Procedure

Qualitative methods were favored in this exploratory study to provide a “thick description” (Geertz 1973) and a better understanding of key actors’ perspectives on YMH collaborative care in a multiethnic neighborhood. A qualitative exploratory approach appeared particularly well-suited, given the lack of research on the viewpoints of migrant youths and their families with regard to community-based YMH collaborative care. The protocol used a transversal (one-time data collection at 6 months following inception of treatment) qualitative multiple case study. It relied on a multi-informant perspective to develop a triangulated, more nuanced understanding based on key stakeholders and allowing for the emergence of both divergent and complementary views. Cases consisted of triads composed of a youth, a parent and a primary care clinician (therapist). This allowed for triangulation of the opinions of the three main stakeholders regarding mental health services for the youth.

### Measures

Between March 2010 and June 2011, semi-structured individual interviews were conducted with all participants (*N* = 15). Informed assent to participate in the study was obtained from the youths, as well as informed consent from the parents and clinicians. Interviews with youths or parents were conducted either at the family’s home or at the CLSC, according to their preference, and in the presence of an interpreter when necessary (one parent interview). Clinicians were interviewed at the CLSC. One co-author with extensive experience in qualitative interviewing conducted all interviews (AJ), each lasting approximately 1 h. The interviews were audio recorded and then transcribed verbatim. The interview with the interpreter included the direct translation of the parent’s narrative into English by the interpreter, which was used for the transcript. The interview guide for the study was built using and adapting Andersen’s (2008) behavioral model of health services use, which includes access, use, efficacy and satisfaction. It included open-ended questions to elicit narratives on the care trajectory, encountered difficulties and explanatory models for such difficulties, the referral process, service accessibility, the treatment process, satisfaction with services and the perceived efficacy of care. The youth interview guide is shown in Table 2; the parent and clinician interview guides targeted the same themes. Probes were used when necessary to explore themes further.

**Table 2:** Interview guide (youth participant)

Key concept	Sample questions
Introduction	-For what reason did you first come to the CLSC?
	-For how long had you been experiencing difficulties?
Explanatory Model	-What did you think was going on in the beginning?
	-What did your family think it was?
Help Seeking Process	*Before coming to the CLSC*
	-When you started having difficulties, did you or your family do something particular or look for help? -If so, what did you do, and who did you go to?
	-What did you think of the help you received?
	-What do you think you needed?
CLSC Services	-When did you come to the CLSC?
	-What happened when you went to the CLSC?
	-Who did you see, and when?
	-What do you think of the services you received at the CLSC? (Or services that were proposed to you)?
Access	-Where would you prefer to have a follow-up? (At the CLSC, at school, in the hospital)
	-Why?
	-Do you feel you were seen quickly enough at the CLSC? -When did you wish to be seen?
	-How is it for you to go to the CLSC (place, time, language)?
Efficiency	-Since you first received services in the CLSC, do you find that your situation and difficulties have changed?-In what way? (Better or worse?)
	-How is your health? -How is it going at school, and at home?
	-Are there things that still preoccupy you?
	-What has helped you the most?-What has been more difficult?
	*At the CLSC*
	-What has helped you the most at the CLSC?
	-Has it changed how you react to difficulties?
	-What was most difficult at the CLSC?
	-Did you receive medication, and what did you think of it?
	-How much were you able to follow the treatment that was suggested?
	-Do you think that people at the CLSC were able to understand you? -Do you think they took into account your whole situation (culture, language, etc.)? -If not, why do you think it was difficult?
Satisfaction	-What did you prefer about the services you received at the CLSC?
	-What would you change to make services better?
Conclusion	-Would you like to add something?

### Data Analyses

Data analysis was conducted following an iterative process favored in qualitative research and using a thematic analysis procedure (Fereday and Muir-Cochrane 2006). Deductive and inductive thematic analysis of all individual interview transcripts was performed by three co-authors (LN, JJL, AJ). This allowed for an iterative coding process including immersion in the interview transcript and outlining a priori and emergent themes (Patton 2002). A priori themes were theory-driven and derived from the semi-structured interview guide. Two researchers read all transcripts from a phenomenological standpoint to detect emergent themes. Another researcher read all transcripts to produce a one-page narrative to recontextualize the themes (Ayres et al. 2003). Transcripts were later coded and thematic agreement discussed by all authors to increase the reliability of the analysis. Transcripts were entered and coded using the specialized software NVivo9. Analysis was done in two steps: first, an analysis was performed looking at the common themes addressed by each category of actor (youth, parent, clinician), to provide a better understanding of the perspective of each type of participant. Second, triads were analyzed as case studies, comparing the narratives, and identifying coherences, contrasts and silences in each subject’s discourse in relationship to those of the other members of the triad. Such procedures allow for analysis triangulation and improve validity (Miles and Huberman 1994). Each step included an iterative process of going back and forth between codes and themes, and the recontextualization of results through re-immersion in the transcripts and the use of narrative summaries.

## Results

### Participants and Interventions

Participants were 15 subjects grouped in 5 triads. Each triad included a youth, one of the youth’s parents, and the youth’s main mental health clinician. Youths were four males and one female, between 12 and 15 years of age. Parents were four mothers and one father. All families were migrants, the youths being either first—or second—generation migrants with at least one parent having migrated. Three families were of South Asian origin, one family was of Southeast Asian origin, and one family was of Canadian and European origin. Primary care clinicians were four females and one male: three social workers, one art therapist, and one psychoeducator. Four youths displayed external behavior problems, while one had a diagnosis of depression and one of attention deficit hyperactivity disorder (ADHD). These are common clinical situations encountered in primary care. All the youths received treatment within a collaborative care setting: they received services from the youth teams at their local CLSCs, which had the support of an on-site child psychiatrist doing direct or indirect consultations (integrated co-location model of collaborative care). Referral to YMH services came either from the youths’ schools (three families) or a family doctor (two).Various modalities of intervention were provided to the families. All received a combination of individual treatment and family intervention, and most families (four) received some form of psychosocial intervention in collaboration with the school and/or youth protection services. Medication was also prescribed for three youths. While all families received part (or, for some, all) of follow-up care at the CLSC, some also had part of the services dispensed at home (three), at school (three), at the hospital (three), or at a youth center (one).

### Thematic Analysis

Results are presented in two sections. The first section describes the most salient themes by actor category. The families’ perspectives (parents’ and youth’s perspective) are presented together as one of the study’s results was that their perspectives emphasized similar themes and therefore benefitted from being considered together, while dissimilarities are also highlighted throughout the analysis. To indicate the level of consensus among actors, the number of people having mentioned a particular theme is put in brackets. One or two out of five is considered low consensus, three out of five, moderate, and four or five out of five high (Palinkas et al. 2007). Case analyses by triads are also presented. This analysis improves validity by recontextualizing the results and providing a deeper understanding of the ways in which key issues are experienced and negotiated by different actors with respect to a specific therapeutic process. It also underscores contrasts between actors’ perspectives and suggests patterns of care trajectories. Participants’ names and sociodemographic information have been disguised to protect confidentiality. Since the majority of subjects were male, all youth are presented as male subjects to ensure further confidentiality as gender issues did not emerge as a salient theme in our results.

### Youth and Parent Perspectives

#### Balancing collaboration, accessibility, and confidentiality

Youth and parent participants acknowledged the primacy of communication between families and clinicians (three youth, five parents) and amongst professionals (one youth, two parents, from separate dyads) to ensure adequate care. Families usually (four) did not initially request services and took a more passive stance, although some later became more active collaborators. Access to interpreters in YMH services for parents non-fluent in official language was noted as instrumental in helping overcome communication challenges (three youth, two parents, twice from same dyad). But participants also emphasized how communication issues posed challenges in terms of confidentiality. They had diverse views regarding the desired balance between communication and confidentiality. This was an especially sensitive issue with regard to collaboration and communication between health services and schools. While some youths (two) and parents (two, same dyad as youth) did not mention concerns about confidentiality issues and could view the possibility of accessing YMH services in school as convenient—even if most of them (four youth, four parents) preferred to be seen at the CLSC or at home—others worried that sensitive information could be leaked in the school environment and have a negative impact on the youth’s or family’s life (two youth, three parent, twice from same dyad). Youths in particular (three) stressed the importance of privacy with regard to school, friends, family or professionals. Families mentioned the fear of stigma if the youth was seen consulting a professional at school. One parent clearly expressed this concern, differentiating between primary school where it might be fine, and high school, where adolescent might feel “embarrassed”. One parent considered collaboration among different professionals involved with a specific youth in various environments as promoting optimal, safe care for the youth, underscoring the benefits of communication to ensure safety, especially in situations in which suicide was a concern. For the parent, this implied the sharing of relevant information between mental health clinicians and school professionals. Interinstitutional collaboration was noted by parents (three) without assessment of its quality.

#### Continuity of people and places

Youths (four) and parents (three) were often imprecise regarding the exact profession of the clinicians they met or the relationships between the different persons involved in their care. Only one parent–youth dyad displayed an excellent grasp of this information, probably due to a familiarity with the system linked to their social position and to one parent having been born in Canada. In contrast with the problems understanding the system of care shown by most of the families, one consistent theme in their discourse was the importance of familiarity and trust within the specific circle of clinicians they encountered (three youth, three parents, twice from same dyad), which often translated into placing considerable importance on the continuity of people and places (e.g., not switching rooms) and the personalization of care (including receiving a warm welcome from the receptionist) in determining the satisfaction with services.

[My wish would be to] continue with the services. And also to make sure that everything is OK afterwards. Not only being there during the crisis, and then to let everything down. […] To have the same person, in the same context, it’s reassuring. […] I think it’s a plus when we’re talking about mental health, when we’re talking about people in distress. […] Actually, it’s essential. (Parent)

Some families and clinicians described years of interventions, and how familiarity and trust was built slowly. Also, comfort with professionals varied among family members, and at times these relationships involved negotiating differences in representations of care in the country of origin and the host country. Youths also placed considerable emphasis on flexibility in the scheduling of meetings.

#### Improvement of communication within the family

Four of the five families (four youth, three parents) mentioned that the services had a positive impact on communication and decreased conflicts within their family. Participants stressed the positive effect on family dynamics as much as the positive outcomes of services for symptoms.

I find that the services are very helpful. It helps us to be able to talk in a suitable manner, correctly, using the right words. As if we can talk instead of insinuating, or being afraid to talk about it, or hiding it, or not knowing what to do with it. I find that it’s really beneficial for my child. (Parent)

Similarly, an adolescent reported how services increased opportunities for family dialogs, allowing him to better understand his parents’ perspectives and feelings:

Once you’re in the meeting, you actually get to communicate with them [your parents] about how you act and you realize how harmful your behavior is. Like that’s what I find useful. And you actually get a chance to realize how your parents feel by their own words. (Youth)

However, discussion of family matters within the clinical space was not consistently seen as positive by youth. The youth cited here, whose difficulties involved externalized problem behavior, first described feeling bored or ill at ease in family meetings, stressing how these discussions should be kept within the family. However, his discourse transformed over the course of the interview to evoke the role of the CLSC in initiating such family conversations and finally to name the positive effects mentioned above. The role of the primary care clinician was further qualified by another parent as “a mediator of common [family] life”, alluding to family conflicts and the importance of having means to resolve them. Despite this emphasis on family intervention, youths (four) also described the beneficial contributions of individual treatment.

### Clinicians’ Perspective

#### Collaboration and institutional support for the clinician

Interprofessional collaboration, interinstitutional partnership and systemic issues were described at great length by clinicians, underscoring both benefits and difficulties of collaborative care, and reporting instances of highly successful partnership and others of less effective collaboration. When commenting on other institutions, clinicians referred mainly to hospitals (two) and youth protection agencies (two), which, with schools, are their main institutional partners. Partnership with schools was discussed mostly in terms of the CLSC’s role as a mediator between families and schools (also mentioned by one parent). Clinicians (four) highlighted positive aspects of collaborative care, including benefiting from the support of colleagues and sharing the burden of complex cases (two). They also mentioned the advantage of being able to consult a child psychiatrist when necessary. Narratives underscored how this co-location model of collaborative care including child psychiatrist availability felt efficient and appropriate and allowed for a best fit when such consultations focused on answering the questions of the referring consultant, offered informal training opportunities for multicultural interventions, and put families at ease. Clinicians (three) also reported instances of less effective collaboration, associated with a lack of knowledge of other institutions’ mandates and communication challenges, leading at times to confusion around other agencies’ roles. The lack of clarity surrounding roles and responsibilities was also mentioned with respect to individual professionals. For example, one clinician, accustomed to working independently in the past, expressed a certain sense of puzzlement brought about by the transition toward a collaborative approach:

It was very unusual for me to work with so many other people. […] I found it very hard to figure out who was supposed to do what. […] I know it is something that the CLSC is still working on, sorting out like what is [each team’s] role. (Clinician)

#### Strengthening the parents’ role and promoting continuity

Like the youths and parents, the clinicians put high value on the work done with parents around family life (five), deploring moments when crisis intervention would get in the way of in-depth exploration of relevant elements of family history or cultural aspects. They also cited the importance of a stable and safe internal service framework, and stressed the need for continuous liaison with families moving between services.

### Case Analysis (Triads)

#### Finding meaning and familiarity

One triad illustrated the initial discomfort of a parent with a care system that uses a different explanatory model from the family, followed by a reconciliation of positions. Jay is a first-generation migrant 14-year-old boy, whose parents separated following family violence. His symptoms of hyperactivity and inattention led the school to refer him to a hospital, which then referred him to a second hospital before he finally came to the CLSC. The boy also displayed sexually inappropriate behavior and episodes of lying. Whereas he had had a trial of psychostimulant medication which was subsequently terminated, the CLSC focused on family intervention and mediation with his school. Both the parent and the boy described a system in which they had primarily been uncomfortable with services. They also acknowledged a very different explanatory model, describing the problem as an academic problem calling for tutoring rather than the ADHD and behavioral problem put forward by the medical system. The mother reported having had difficulties navigating the system and differences in points of view, yet she described being satisfied with services, mentioning the importance of follow-up care which had reconciled her position with the clinician’s. The clinician had in fact sought to develop a comprehensive understanding of the situation including cultural aspects, sensing the differences in understanding that were acting as a hindrance to the therapeutic relationship.

A second triad reflects a simpler consultation process characterized by similar explanatory models being held by the family and service providers, and by a certain ease in negotiating the system. Florian is a 13-year-old boy living with his parents, a mixed couple of Canadian and European origin. His mother felt he was too withdrawn socially and that the communication between Florian and his father was arduous. He had previously been diagnosed with ADHD and started on medication. Given the non-optimal use and effect of the medication, the family doctor referred Florian to the local services center to explore other ways of working through his difficulties. Over the past 2 years, he had been seeing a social worker for individual therapy, with occasional family sessions as well. Florian’s mother appreciated the possibility of collaboration between the doctor, the CLSC and the school and described the benefits of the family approach, but still worried around her son’s depressive symptoms. Florian described his problems in the same words as the clinician and considered the combination of medication, individual therapy and family work to be quite beneficial. He also thought the setting for the therapy adequate. The clinician expressed satisfaction with the boy’s outcome and the family’s collaboration, described the need to mediate the mother’s anxiety, and mentioned how easily this family had negotiated the health system.

#### Comfort with services as part of access to care

The following triad shows how comfort with services developed over the course of the trajectory within the care system, for both the youth and the parent, can facilitate adherence to treatment. Marco’s on-going care started when he was in primary school. He first saw a social worker in his French-speaking school. When the social worker went on maternity leave, she transferred him to an English-speaking CLSC co-worker. This coincided with the change from primary school to high school. Marco comments:

I used to get mad and pissed off really easily so Sophie [social worker] would help me calm down. […] At the beginning I thought that it was weird and I was uncomfortable, but as we went on it was easier […] because we got to know each other. […] Everything was going to be okay because she said it was confidential. […] She was like a best friend. […] Well, when I met her at school it was not really her place; it was really the nurse’s office […]. But when I go see Lewis at the CLSC, it is his place […]. It feels more of a place to talk instead of a nurse’s office. […] At school, there is always people coming in and out. Sometimes it was not always confidential […][Sophie] said that I could go to see her anytime. So, sometimes, in class when I did not feel well I would go there. But now in high school, I have to wait all the way until after school before I can go see Lewis. […] When I spoke to Sophie and Lewis it is like I got lighter. It is like something came off my back and it feels better. […]I would tell [other youth coming to the CLSC] not to worry, everything there is confidential. (Youth)

The mother explained she had had to be proactive in order to have her son’s difficulties recognized by the school and to obtain services. Once the services were offered, the process went smoothly and the mother felt comfortable with the process. She also mentioned a child psychiatry consultation at the CLSC as a turning point: the father had attended and the family dynamics had been explored with respect to the immigration process. This permitted a challenging situation to come to light and fostered further positive changes in the family. The mother also spoke of her familiarity and comfort with the CLSC as the place where her children were vaccinated in early childhood, and which had provided them with the opportunity to go to summer camp.

#### Negotiation of values and services by first-generation migrant families

The family in the next triad struggled with authority and confidentiality issues when confronted with different values in the host society. John’s mother spoke of her difficulties with her children, linking these difficulties to the challenge of negotiating authority within the migratory process. At the same time, she explained her pathway through care, spoke of her own sense of feeling dispossessed of her authority at times, and stressed how services and society contributed to making her fragile as a parent, while describing how she regained a sense of power throughout the services offered:

The main thing is they [my sons] were not listening to me. […] I cannot handle it. […]. So I asked the doctor, who referred to a social worker. We found out something that happened before with my kids […] each one had an educator and that was helpful […] but I had a disadvantage.[…] The [social worker] always told [my youngest son]: if there is something at home or something happens you can let us know. […] He [my son] thought he could do whatever he wanted. […] He was too young for that. […] I still have to decide. […] They [my children] were already out of control and now it is more out of control. […] He would yell at me: “F[…] immigrant why are you talking to me like this?” […] He said: “I am a citizen!” And I said: “No you are not!” […] It was not only the social worker— it was everywhere, the school also. […] [In] my country, never ever did we question our parents. [Here] I think there is a lot of violence so that is why they have those rules to get permission from the children. (Parent)

This struggle was further complicated by confidentiality issues: the mother felt her previous therapist had breached confidentiality when she informed the school that “family had something that was going on”. The mother said: “She [the therapist] should know how delicate these things are”. The mother went on to describe her sense of disempowerment when her son was referred to a detox center which she was told was totally confidential given that the child was over 14.

I had no right to have any information. […] Parents don’t have freedom, no rights. He is only fifteen and I don’t have rights for my son to find out when his treatments are! […] In my country, even at age eighteen or age twenty we listened to our parents. (Parent)

The mother changed therapists and the new clinician explained that the latter’s role was to “improve [family] relations and for the [mother] to be able to reclaim some of her authority”. The mother commented:

Because [the second therapist] she is old, […] she keeps it in mind [to tell children to obey their parents if the parents are caring]. She is thinking like my country. […] Now they [my sons] do not speak like this [in a derogatory manner]. (Parent)

In fact, the second clinician described how a dialog with the family about their country of origin and religion became an entry point for the family to share extensively with her. Interestingly, the youth also commented on the positive aspects of the therapist taking the side of his mother at times and his side at other times, and of the benefit of the parent’s role over the child [authority]. He felt that, overall, his family had gotten closer:

I think they [clinicians] [should] talk with the mother first, which is a really good idea, rather than just talk with the child itself. Because [the] child doesn’t have a perfect memory; he can forget. (Youth)

## Discussion

Our pilot study offers avenues to better understand the important challenges in offering appropriate mental health care to vulnerable families (Nadeau et al. 2012). Results support the importance of collecting multiple informant perspectives when addressing the quality of mental health care. Interesting similarities and contrasts can be identified when looking at the perspectives of different study stakeholders. Our results based on the thematic analysis by actor category suggest that migrant youth and their parents are particularly concerned with YMH care settings and how comfortable they are with such venues, as well as with the integration of family issues into treatment. Clinicians emphasize the collaborative aspect of care while focusing on its systemic context. They address the importance of interprofessional and institutional support, while underscoring the need to understand family vulnerability and any sociocultural factors influencing care. The triad results provide further support for the importance of the care setting while adding emerging themes that can be viewed as part of the overarching framework of care (exploring differences between explanatory models held by family and clinicians, addressing cultural difference in values, and observing the evolution of trust within the therapeutic process). Based on these results, four themes emerged that should be further researched as potential elements of an optimal care setting for collaborative YMH services for migrant families: (1) establishing an equilibrium between communication, collaboration and privacy/confidentiality, (2) special attention to ensuring the continuity of care and the creation of a welcoming environment where trusting relationships can develop, (3) the inclusion of family intervention, and (4) the provision of collaborative decision-making pathways to care, addressing interprofessional and interinstitutional collaboration as well as cultural differences in explanatory models and values.

Adapted communication appeared in our results to be one of the crucial ingredients in pathways to care. A judicious use of communication between professionals is a necessity in models of collaborative care as partnership implies the sharing of information and discussion of clinical situations. However, there is little talk in collaborative care of the benefits of discussing interprofessional communication—even when framed by confidentiality and privacy protocols—with families to preserve the latter’s sense of comfort. This issue seems particularly salient in YMH, where clinical situations often involve a few, if not a wide range of professionals, as well as challenges regarding privacy. Our results suggest that families are particularly sensitive to this issue, and that there is a benefit of communication modalities to be adapted to each clinical situation to foster a sense of optimal collaboration, safety and confidentiality. Further research could explore how an acknowledged consensus around communication regarding the content of the shared information and the terminology to be used when describing the youth’s issues could be helpful in establishing the right balance between knowledge-sharing and the protection of privacy, and how this consensus could further benefit from being reviewed over time, as preoccupations of families vary throughout the care process. It also seems important that schools, which are key partners, be given special consideration: while schools represent an open setting that may offer a great deal of support, they also constitute an environment in which young people’s stories or difficulties may be more exposed. Although schools have confidentiality protocols with clear rules around accessibility to privileged information concerning children, students and their families may fear a breach in confidentiality and indiscretion, as our results suggest. If mental health services are offered directly on the school premises, it adds the benefit of proximity. However, offering services in schools, especially high schools, may make it difficult to hide the fact that a youth is seeing a psychologist or any other professional. Trust in the intervention provided builds on belief in the confidentiality of the system and familiarity with the system. For migrant families who are unfamiliar with the host society’s different institutions and who may have experienced marginalization or discrimination, the building of trust may require extra steps. Our results tend to support that providing a safe clinical environment also means helping youth and parents learn about and get their bearings within the unfamiliar care system.

Our results also suggest benefits to building a sense of continuity of services that can be materialized in efforts to keep a family with the same clinician or same team, and to facilitate liaisons between institutions. They further tend to show that it can be challenging for clinicians to develop a trusting relationship and alliance with migrant youth and families who have already experienced a number of ruptures in their migratory process. While this favors a certain stability of clinicians within teams, the reality of collaborative care often means that patients circulate between services and institutions. A meta-summary by Haggerty et al. (2013) shows that the experience of continuity of care when patients see multiple clinicians contributes to patient security and confidence. According to the authors, this translates into the patient being supported through transitions as well as having one trusted clinician who views the patient as a partner while helping the patient navigate the patient’s care pathway in the health care system. As illustrated by our results, it should be further explored how, in front of such mobility in the health care system, the creation of a safe space could be established in YMH in collaboration with all involved institutions. The exploration of ways to foster a sense of continuity and safety appears particularly important when working with migrant families for whom the host society’s health system may feel quite unfamiliar. Within such a study the aforementioned optimized communication could be one of the ingredients considered in fostering continuity of care for migrant youth and their families.

Other potential ingredients of a safe care environment pertain to the creation of a welcoming environment. Youth in particular stressed how details affecting their degree of comfort with therapy rooms and the sense of welcome they experienced from the people with whom they first came into contact in the system of care influenced how they felt about services overall. Further research could examine the potential benefit in terms of accessibility of care for community-based health institutions to place greater importance on ensuring a welcoming therapy setting (people, space and timeframe) for youth and families. A safe care environment ties in with the concept of cultural safety, in the sense of a culturally adequate and respectful setting. The concept of cultural safety was developed in New Zealand to describe how accessibility to health services for Indigenous people meant an environment where they would feel welcomed and not discriminated against (Smye and Brown 2002). The concept is echoed in the literature devoted to migrants (Nadeau et al. 2014), and the current study’s narratives again underscore the positive effect of a safe care environment.

Our results further propose high consensus among families and clinicians regarding the importance of family interventions. Families’ narratives stress the crucial effect of such interventions and the positive impact of clinical spaces in which youths and their parents felt sufficiently at ease to dialog. These narratives place the family relationships at the heart of both the problem and the solution. The results raise questions about models of care that focus mainly on the young person, and in which confidentiality and autonomy (the individual perspective of the youth) issues are put in the forefront while parents’ contributions are left in the background. Some such models involve parents only when the youth is at risk. Although the narratives of our research participants highlight the importance of privacy and individual intervention, they also suggest the important benefits of models that address communication between youth and parents within the clinical space in situations involving externalized behavioral issues or internalized symptoms. They underscore the complementarity of interventions that address symptoms (psychoeducational strategies) with those that target family dynamics. Clinical outcomes are often measured in terms of change in symptomatology (e.g., as measured with a grid such as a depressive inventory) and impairment. Our results suggest the importance of improvement in family communication (with work on cohesion and conflict), which in fact may be both an important outcome and mediating factor to measure. Whereas the literature on systemic and family therapy has regularly stressed this aspect, the principal current discourse on mental health strategies often emphasizes medical and individual therapies instead (Dunnachie 2007). Our results echo other research suggesting that the association between symptoms in adolescents and family functioning is a circular process wherein each factor affects the other over time (Brière et al. 2013).

Lastly, developing a clear and collaborative decision-making pathway was important to participating clinicians offering YMH services to migrant patients and their families. In YMH interinstitutional collaborative care, service effectiveness depends on productive communication channels (Chang et al. 2014), and on the capacity of partners to complement one another in an optimal way to arrive at the best decisions regarding treatment. Our results suggest that these elements of partnership concern both institutions and individuals within the institutions, in terms of familiarizing themselves with one another’s roles, adopting a collaborative attitude characterized by confidence in one another. The results also propose the importance of providing space for clinical discussions. Previous research has shown how forums for clinical discussions are instrumental in building collaborative care (Nadeau et al. 2012). Yet this is an important challenge for health and social services institutions under considerable pressure due to budget cuts.

Our results further suggest that treating families and young people as partners in care to improve access to services and participation in decision-making implies understanding their point of view and being open to taking their explanatory model of the problem into consideration in the dialog around treatment. A study by Ødegård and Bjørkly (2012) describes how the fit between the explanatory models of parents and those of clinicians can be an important factor in outcomes. One possible point of divergence between families and clinicians may be over the values to be transmitted to youth. This includes attitudes toward authority, which is a fundamental value and object of negotiation in families when children reach adolescence, and probably even more so when immigration transforms its representations. Our results suggest that, in families struggling to empower themselves when dealing with the mental health system, migrant families face the extra challenge of negotiating this authority from a particularly vulnerable position linked to their unfamiliarity with the system and to the way the system perceives migrants, which is all too often as less capable. Furthermore mental health issues or palpable tensions within the parent–child relationship may produce ambivalence and challenges with respect to the role of partners in care. These results thus propose that making families partners in decision-making involves allowing both parents and youth a form of autonomy and self-agency within the system of care while providing room for generational differences, and supporting them as true partners. Our results also put forward how the youth and parents’ perspectives on decisions and therapy may transform throughout the process of treatment, with some moving from a passive stance to an active one. This suggests that taking into account their perspectives regarding treatment implies interpreting their voices in their proper context, keeping in mind that the presence of mental health issues in families may weaken their ability to reflect optimally on finding a solution. Patient preferences are considered an integral part of evidence-based practice (McHugh et al. 2013), yet they have to be assessed in a longitudinal manner that acknowledges their potential transformation over time.

This study has limits inherent to pilot studies. The generalizability of our results is restricted by the small sample size and specific research context, by the transversal nature of the data, as well as by the recruitment strategy. A sample of 5 triads and 15 participants provides non-optimal saturation of data. Some of our results are likely to be influenced by the characteristics of the neighborhood and community clinic (including the type of proximity services offered) in which the research was conducted and this limits the capacity to generalize to other contexts. Given the pilot project nature of the study, only transversal data were collected, rather than longitudinal data which could have provided a more nuanced interpretation. Moreover, for ethical reasons, we relied on clinicians to approach families for the study. Clinicians were invited to identify and approach youths who might be potential participants before a member of the research team contacted them to explain the study and invite them to participate. While this offered the ethical advantage of ensuring families and youths would be comfortable with the study, in addition to facilitating the alliance, it also restricted recruitment, resulting in the sample not being fully purposeful. Furthermore, this sample included more externalizing symptoms than internalizing ones, and was comprised mostly of Asian subjects (from South-East Asia and South Asia). While participants seemed representative of migrant youths in the administrative region of the community-based services, further research should look at a more comprehensive sample in terms of symptomatology and ethnic diversity to determine whether the results would then be replicated or transformed. However, while the small sample does not allow for generalization to other contexts, this study on YMH services offers a perspective rarely explored in the literature, taking into account the experience of youths, parents and clinicians, and has provided useful hypotheses for the development of a more substantial research project on YMH services for migrant families that would also benefit from a mixed-method longitudinal design. This type of design would allow examining the hypotheses more rigorously, with both qualitative and quantitative instruments (for example to look at importance of family dynamics through semi-structured interviews as well as through a validated questionnaire). Another limitation is the absence of a comparison group of non-migrant youth. To a certain extent, the results may also reflect preoccupations regarding YMH care shared by non-migrant families. This again underscores the necessity of conducting a larger research project to examine these issues more globally with both migrant and non-migrant subjects.

## References

[CR1] Andersen RM (2008). National health surveys and the behavioral model of health services use. Medical Care.

[CR2] Aupont O, Doerfler L, Connor DF, Stille C, Tisminetzky M, McLaughlin TJ (2013). A collaborative care model to improve access to pediatric mental health services. Administration and Policy in Mental Health and Mental Health Services Research.

[CR3] Ayres L, Kavanaugh K, Knafl KA (2003). Within-case and across-case approaches to qualitative data analysis. Qualitative Health Research.

[CR4] Brière FN, Archambault K, Janosz M (2013). Reciprocal prospective associations between depressive symptoms and perceived relationship with parents in early adolescence. Canadian Journal of Psychiatry.

[CR5] Chang ET, Wells KB, Young AS, Stockdale S, Johnson MD, Fickel JJ, Jou K, Rubenstein LV (2014). The anatomy of primary care and mental health clinician communication: A quality improvement case study. Journal of General Internal Medicine.

[CR6] Dunnachie B (2007). Evidence-based age-appropriate interventions: A guide for child and adolescent mental health services (CAMHS).

[CR7] Ellis BH, Miller AB, Baldwin H, Abdi S (2011). New directions in refugee youth mental health services: Overcoming barriers to engagement. Journal of Child & Adolescent Trauma.

[CR8] Fereday J, Muir-Cochrane E (2006). Demonstrating rigor using thematic analysis: A hybrid approach of inductive and deductive coding and theme development. International Journal of Qualitative Methods.

[CR9] Gampetro P, Wojciechowski EA, Amer KS (2012). Life concerns and perceptions of care in adolescents with mental health care needs: A qualitative study in a school-based health clinic. Pediatric Nursing.

[CR10] Garland AF, Brookman-Frazee L (2015). Therapists and researchers: Advancing collaboration. Psychotherapy Research.

[CR11] Geertz C (1973). The interpretation of cultures: Selected essays.

[CR12] Haggerty JL, Roberge D, Freeman GK, Beaulieu C (2013). Experienced continuity of care when patients see multiple clinicians: A qualitative meta-summary. Annals of Family Medicine.

[CR13] Kates N, Mazowita G, Lemire F, Jayabarathan A, Bland R, Selby P (2011). The evolution of collaborative mental health care in Canada: A shared vision for the future. Canadian Journal of Psychiatry.

[CR14] Kazdin AE, Holland L, Crowley M (1997). Family experience of barriers to treatment and premature termination from child therapy. Journal of Consulting and Clinical Psychology.

[CR15] Kleinman A (1988). Rethinking psychiatry: From cultural category to personal experience.

[CR16] Kolko DJ, Campo JV, Kilbourne AM, Kelleher K (2012). Doctor-office collaborative care for pediatric behavioral problems: A preliminary clinical trial. Archives of Pediatrics & Adolescent Medicine.

[CR17] Leckman JF, Leventhal BL (2008). Editorial: A global perspective on child and adolescent mental health. Journal of Child Psychology and Psychiatry.

[CR18] Lipton H, Donsky A (2012). Healthy minds/healthy children outreach service: Lessons learned after eight years. Journal of the Canadian Academy of Child and Adolescent Psychiatry.

[CR19] McGorry P, Bates T, Birchwood M (2013). Designing youth mental health services for the 21st century: Examples from Australia, Ireland and the UK. The British Journal of Psychiatry.

[CR20] McGuirk J, Button S (2013). Commentary: Improving children’s services: Building partnerships between providers and researchers. Administration and Policy in Mental Health and Mental Health Services Research.

[CR21] McHugh RK, Whitton SW, Peckham AD, Welge JA, Otto MW (2013). Patient preference for psychological vs pharmacologic treatment of psychiatric disorders: A meta-analytic review. The Journal of Clinical Psychiatry.

[CR22] Miles MB, Huberman AM (1994). Qualitative data analysis: An expanded sourcebook.

[CR23] Nadeau L, Measham T (2006). Caring for migrant and refugee children: Challenges associated with mental health care in pediatrics. Journal of Developmental & Behavioral Pediatrics.

[CR24] Nadeau L, Jaimes A, Rousseau C, Papazian–Zohrabian G, Germain K, Broadhurst J, Battaglini A, Measham T (2012). Partnership at the forefront of change: Documenting youth mental health services transformation in Quebec. Journal of the Canadian Academy of Child and Adolescent Psychiatry.

[CR25] Nadeau L, Rousseau C, Measham T, Kirmayer L, Rousseau C, Guzder J (2014). Addressing cultural diversity through collaborative care. Cultural consultation.

[CR26] Obeyesekere, G. (1990). *The work of culture: Symbolic transformation in psychoanalysis and anthropology* (Vol. 1982). Chicago: University of Chicago Press.

[CR27] O’Cathain A, Murphy E, Nicholl J (2010). Three techniques for integrating data in mixed methods studies. British Medical Journal.

[CR28] Ødegård A, Bjørkly S (2012). The family as partner in child mental health care: Problem perceptions and challenges to collaboration. Journal of the Canadian Academy of Child and Adolescent Psychiatry.

[CR29] Palinkas LA, Criado V, Fuentes D, Shepherd S, Milian H, Folsom D, Jeste DV (2007). Unmet needs for services for older adults with mental illness: Comparison of views of different stakeholder groups. The American Journal of Geriatric Psychiatry.

[CR30] Patton MQ (2002). Qualitative research and evaluation methods.

[CR31] Pina AA, Gonzales NA (2014). The role of theory and culture in child and adolescent prevention science: Introduction to the special section. Journal of Clinical Child & Adolescent Psychology.

[CR32] Pumariega AJ, Rothe E, Pumariega JB (2005). Mental health of immigrants and refugees. Community Mental Health Journal.

[CR33] Rechtman R (2000). Stories of trauma and idioms of distress: From cultural narratives to clinical assessment. Transcultural Psychiatry.

[CR34] Rousseau C, Ammara G, Baillargeon L, Lenoir-Achdjian A, Roy D (2007). Repenser les services en santé mentale des jeunes: la créativité nécessaire [Rethinking youth mental health services: A call for creativity].

[CR35] Rousseau C, Measham T, Nadeau L (2013). Addressing trauma in collaborative mental health care for refugee children. Clinical Child Psychology and Psychiatry.

[CR36] Smye V, Brown AJ (2002). “Cultural safety” and the analysis of health policy affecting Aboriginal people. Nurse Researcher.

[CR37] Ter Kuile S, Rousseau C, Muñoz M, Nadeau L, Ouimet M-J (2007). The universality of the Canadian health system in question: Barriers to services for immigrants and refugees. International Journal of Migration, Health and Social Care.

[CR38] Tylee A, Haller DM, Graham T, Churchill R, Sanci LA (2007). Youth-friendly primary-care services: How are we doing and what more needs to be done?. The Lancet.

